# We Need Compassionate Leadership Management Based on Evidence to Defeat COVID-19

**DOI:** 10.34172/ijhpm.2020.73

**Published:** 2020-05-10

**Authors:** Agnes Binagwaho

**Affiliations:** University of Global Health Equity, Kigali, Rwanda.

**Keywords:** COVID-19, Implementation Science, Implementation Research, Evidence-Based Decisions

## Abstract

The current pandemic of coronavirus disease 2019 (COVID-19) has had unprecedented reach and shown the need for strong, compassionate and evidence-based decisions to effectively stop the spread of the disease and save lives. While aggressive in its response, Rwanda prioritized the lives of its people – a human right that some governments forget to focus on. The country took significant steps, before the first case and to limit the spread of the disease, rolled out a complete nationwide lockdown within one week of the first confirmed case, while also providing social support to vulnerable populations. This pandemic highlights the need for leaders to be educated on implementation science principles to be able to make evidence-based decisions through a multi-sectoral, integrated response, with consideration for contextual factors that affect implementation. This approach is critical in developing appropriate preparedness and response strategies and save lives during the current threat and those to come.

The coronavirus disease 2019 (COVID-19), having now spread to 211 countries and territories, has created a dramatic situation throughout the world, unveiling fractures in systems that prevented an efficient response to this pandemic.^1^ It is identified as the most significant pandemic faced by humanity in the past 100 years, not because of the number of deaths it has caused – remember that AIDS has already killed 32 million people – but because of its rapid spread and the unprecedented economic damage and global lockdown it has generated.^2^

Over the last five months, we have witnessed leaders repeatedly contradicting themselves in their response to COVID-19.^3^ When China, the first country affected, took severe social distancing measures and restrictions aimed at halting the alarming trajectory that COVID-19 was taken, the country was heavily criticized by the Western world.^4^ It was only months later when the virus began taking a similar toll in their own countries that leaders in the United States and Europe changed their views and policies.

Unlike China, Europe and the United States, now the epicenter of the pandemic, have recent, prior examples of the devastating consequences of COVID-19, as well as proven methods and guidelines from international health agencies such as the World Health Organization (WHO) and Centers for Disease Control (CDC) to help them prevent unnecessary infection and deaths.^1,5,6^ This wealth of information should have informed all countries about the risks. Thus, their lack of preparation and inability to implement the required actions to contain the spread are difficult to defend with excuses.

This pandemic has had an unprecedented reach and requires commitment and changing attitudes at the highest levels of leadership in all countries, similar to a world war against a common enemy. This means implementing unprecedented governmental decisions at the first sign of this enemy, to limit spread and protect its citizens, albeit dramatically shifting life as we know it. Countries that have failed to exercise this level of leadership, whether due to politics or a failure to consider the virus as a threat, have done a serious disservice to their own countries and to the world, allowing preventable suffering to take place because of their misjudgement.

From my perspective, my own country, Rwanda, a country of 12.5 million people located in sub Saharan Africa, has had a compassionate, strong response to this pandemic.^7^ To protect our people, we implemented national policies around COVID-19 prevention and response beginning in mid-January, with all travellers arriving at a Rwandan airport screened and all suspected cases tested.^8,9^ And, when the first COVID-19 case was diagnosed in Rwanda in mid-March, the government rapidly responded without waiting for a second case to emerge. Immediately, systematic contact tracing and testing were conducted, and the country implemented a national lock-down the following day. This included closing schools and religious spaces, restriction of all non-essential travel within the country, and closing the borders.^10^ Within one week, all incoming and outbound commercial flights were suspended, with only cargo entering with goods necessary for the population.^11^ The measures were mandatory and enforced, and the vast majority of the population complied. This public response is not surprising; according to the Wellcome Trust Global Monitor Report, Rwanda has the highest levels of population trust in the health sector in the world.^12^ The country and its people have also learnt from past experiences in implementation, action and behaviour in successfully preventing Ebola from entering into our borders.

These measures may sound extreme, and unrealistic, especially in a developing country with a relatively small number of cases compared to other settings, and which face, like almost all countries, a shortage in test and protective tools for health workers, but this could not be further from the truth. Our leadership thoughtfully considered the costs of inaction. The cost of not implementing these measures and risk of suffering far exceeded the concerns about the economic toll that the country would endure.

Rwanda approached this decision as an investment into its greatest resource: its people. The government also recognized the impact the lockdown had on the vulnerable. To ensure that all those in need received food assistance, Rwandan leadership forfeited one month of salary, while freeing up a budget and raising funds within communities.^13^ Our government did not hesitate to do what was right to protect the lives of its people – a human right that governments worldwide must hold dear both in word and in action.

Another major global failure throughout this pandemic, on the part of many leaders, is that knowledge from the past is not being effectively translated into action. Should not we have learned more by now, after the pandemics the world has faced throughout history, most recently with H1N1 influenza, Ebola, and Zika? The fact that we have so ineffectively applied these lessons in some countries, such as the importance of contact tracing, from these outbreaks fully demonstrates that the world is suffering from a literacy problem in which we refer to as “implementation science.”^14^ Failure to learn such critical lessons from the past is a failure of leadership.

The ineffective leadership to stop the spread of COVID-19 has highlighted the need for leaders to learn more about the principles of implementation science in order to prepare appropriate preparedness and response strategies and plans and be able to make difficult decisions in the face of uncertainty in order to save lives. Implementation science provides us with an arsenal of tools that government leaders should be using to shape the response needed to protect the population against this unique situation in any country. These tools help us to effectively assess the local, national, regional and international contexts that should influence the current actions and how to shape an evidence-based response to even unknown threats.^14^ It also can guide the coordination of a response in a multi-sectoral and integrated manner, to ensure good communication and the provision of quality services. Embracing an implementation science approach – one that creates synergy, solidarity, and trust in shaping the response – is needed during such challenging times.^14^

The COVID-19 pandemic we face today alerts us that it is not only about the level of sophistication of the health sector, nor the wealth of a nation that makes it successful in managing a public health crisis. Rather, it is about the courage and ability to rapidly implement tough solutions that are evidence-based. It is this courage, compassion, and evidence-based decision making that we need in our leadership to manage the current threat and those to come.

## Ethical issues

Not applicable.

## Competing interests

Author declares that she has no competing interests.

## Author’s contribution

AB is the single author of the paper.
